# Prevalence of school related violence in seven countries: A cross-sectional survey

**DOI:** 10.1371/journal.pone.0301833

**Published:** 2024-05-15

**Authors:** Ariel BenYishay, Rachel Sayers, Jessica Wells

**Affiliations:** 1 Department of Economics, William & Mary, Williamsburg, Virginia, United States of America; 2 AidData, William & Mary, Williamsburg, Virginia, United States of America; Caleb University, NIGERIA

## Abstract

Violence against children in schools harms the affected children, limits their learning and educational attainment, and extends its harms to families and the broader communities. However, to date, comparable cross-country data on violence against children in schools has not been available. We utilize the Violence Against Children and Youth Surveys (VACS) to estimate school-related violence against children in seven countries (Honduras, Kenya, Malawi, Nigeria, Tanzania, Uganda, and Zambia). Leveraging the unique comparability of the surveys, we are able to estimate both physical and sexual violence experienced in childhood and adolescence among youth aged 13-24. Where possible, we also disaggregate by gender and perpetrator type. Overall, within our sample seven countries, we find that 12.11-44.63% of females and 14.28-53.85% of males experienced at least one form of violence. Males experience higher levels of school-related violence and a significant portion of this is due to experiencing physical violence perpetrated by male classmates.

## Introduction

The United Nations Sustainable Development Goals (SDGs) “recognize that ending poverty and other deprivations must go hand-in-hand with strategies that improve health and education, reduce inequality, and spur economic growth” [1]. SDG4 and SDG5 work together to ensure education opportunities for all and to empower women and girls. One barrier to achieving these goals is school-related violence. Children who experience violence in schools suffer a variety of negative educational impacts, including lower test scores and higher rates of absenteeism [2, 3]. In addition, the World Bank estimates that these impacts lead to subsequent losses in lifetime earnings that exceed $11 trillion [4]. The detrimental consequences of school-related violence extend to society as a whole.

However, to date, estimates of the prevalence of school-related violence have been limited to small-scale samples or relied on modeling of these rates. A few studies have estimated either the country-specific prevalence or location specific prevalence of related forms of violence such as violence perpetrated by peers, the prevalence of violence against disabled children in school, and the prevalence of childhood violence, including violence at school [5–7]. One study by Devries et al. produces “the first age-specific and sex specific prevalence estimates by perpetrator type for physical, sexual and emotional violence against children globally” [8]. This particular estimate was produced by synthesizing 643 studies across 171 countries. Other studies focus on the impacts of school-related violence but do not generate broad prevalence estimates for this violence. These include analyses of the impacts of various forms of school-related violence including bullying on health outcomes, physical violence on mental health and education performance, and childhood violence on educational outcomes [2, 9, 10]. This work is also limited to non-representative samples drawn in particular settings, frequently limited to district or subnational region scales. Drawing broad, reliable inferences on the prevalence of violence in schools across many settings has thus not been possible, constraining our ability to understand the severity of school-based violence.

An additional dimension of school-related violence is its intersection with gender-based dynamics. An emerging set of research focuses on school-related gender-based violence (SRGBV). SRGBV is “any act or threat of sexual, physical or psychological violence occurring in and around schools, perpetrated as a result of gender norms and stereotypes, and enforced by unequal power dynamics” [11]. However, understanding the extent and prevalence of this type of violence within a particular country is challenging because few surveys have addressed these issues, and these have generally covered small, localized samples. Again, drawing broad scale inference about the prevalence of this violence remains challenging.

In the absence of such reliable, cross-national measures, many policy discussions have instead relied on estimates based on assumptions and modeling. For example, the United Nations estimates that 246 million children across the world are affected by school-related violence each year, but this estimate is based only on a 2006 UN Study on Violence against Children that reported 20-65% of schoolchildren as being affected by verbal bullying, combined with an estimate of the total number of children in school on any given day around the world [12]. UNESCO’s 2011 Global Education Digest report estimates there were 1.23 billion children are in primary or secondary school on any given day [13]. While such global estimates remain critical for policy, they must be built or validated by robust, multi-country sample surveys.

In this paper, we overcome the challenges of estimating the extent of school-related violence and understanding the types of violence that males and females experience. Our study not only allows us to understand the types of violence respondents experience but also at whose hands they experience it. We do this by assessing violence across multiple countries utilizing the Violence Against Children and Youth Surveys (VACS). The VACS explore various forms of physical, emotional, and sexual violence against children and young adults and include important information about perpetrators [14]. To date, the VACS have been carried out in 24 countries, and several countries have conducted more than one round. Country reports summarizing findings from the VACS are published for each survey round, and an array of academic studies have assessed the prevalence of violence against children but none of these reports have addressed school-related violence to date [15–21]. Given the paucity of country-specific data on school-related violence and its gendered dynamics in particular, nationally representative quantitative academic research has been limited. We use information that VACS respondents report on the type of perpetrator for each incident to focus on the prevalence of physical and sexual violence in schools across seven countries. This methodology allows us to create consistent prevalence estimates across multiple country survey waves, thereby drawing inferences about this prevalence that are more broadly generalizable. We also assess the extent of variation in this prevalence both across countries and child demographics.

We find that many children experience physical violence at school, much more than might be commonly believed or otherwise reported. The overall prevalence estimate of school-related violence, including sexual and physical violence, shows that 33.7% of females and 33.8% of males experience this type of violence. Secondly, we find that, although male and female classmates perpetrate violence against opposite-gender classmates, this occurs less frequently than same-gender violence. Lastly, we also find that while the prevalence of sexual violence is lower than physical violence, this still occurs and is perpetrated against male and females.

This paper is structured as follows: (1) in the Methods section, we review the VACS data utilized in this study in more detail and highlight the perpetrator categories we analyze; (2) in the Results section, we present salient findings about school related violence; (3) in the Discussion section, we discussion the implications of our findings; (4) we then provide concluding remarks in our last section.

## Methods

This section reviews the data for our study and outlines the empirical strategy utilized to determine the prevalence of school-related violence across the seven country survey waves (Honduras (2017), Kenya (2010), Malawi (2013), Nigeria (2014), Tanzania (2009), Uganda (2015), and Zambia (2014)). Our analysis is designed to identify school-related violence; estimate its prevalence; and disaggregate by sex, violence categories, and types of perpetrators.

The VACS, led by the U.S. Centers for Disease Control and Prevention and in partnership with Together for Girls, are nationally representative surveys of children and young adults ages 13 to 24. The surveys are designed to measure the prevalence (number and percentage) and circumstances surrounding various forms of violence, including physical and sexual violence, against males and females in childhood, adolescence, and young adulthood. Importantly, these surveys provide valuable information about the type of violence experienced, the perpetrators (including teachers and classmates), and the types of services accessed by victims after the violence. Due to this unique survey structure, we can distinguish school-related violence from violence happening in other contexts.

While some differences arise across countries, the sampling frame was drawn from the most recent census and the primary sampling units (PSUs) were the enumeration areas (EAs) from that census round. The samples produced for each of the countries are designed to be nationally representative. Oversampling was done in some countries around areas of interest such as location or HIV status. The VACS utilize a three-stage cluster sample survey design. For each country, at least 100 EAs were randomly selected but the exact number varies by country. A split sample approach was utilized to allow for separate estimates of male and female violence victimization. To accomplish this, the female surveys were carried out in separate EAs from where male surveys were conducted which ensured the confidentiality of respondents and eliminated the risk that the perpetrator and victim were both interviewed in the same community. After selecting the EAs, a team mapped and listed all of the housing structures in that area. Using that list, a select number of households were randomly selected for the survey. From there, one member of the household was randomly selected from all eligible household respondents aged 13-24 years old. Country specific parameters utilized in the sample selection as well as other survey details can be reviewed in each of the VACS methodology paper and country specific reports [14, 15].

We include Honduras, Kenya, Malawi, Nigeria, Tanzania, Uganda, and Zambia in our analysis and Table 1 shows a side-by-side comparison of several violence estimates. The table shows the prevalence of different types of violence experienced prior to age 18 by gender and among those aged 18-24 at the time the survey was administered. The first seven columns are the countries included in our school violence analysis while the other 10 countries are geographically located in Central America and Africa. We see that the countries included in our school violence analysis have similar prevalence estimates of violence when compared to the broader sample.

**Table 1:** Prevalence of different types of violence and multiple forms of violence experienced prior to age 18 by gender, 18-24.

	Respondent Category	Honduras	Kenya	Malawi	Nigeria	Tanzania	Uganda	Zambia	Botswana	Colombia	Cote d’Ivoire	El Salvador	Haiti	Lesotho	Mozambique	Namibia	Rwanda	Zimbabwe
		2017	2010	2013	2014	2009	2015	2014	2015	2018	2018	2018	2012	2018	2019	2019	2015	2017
Sexual violence only	Female	4.9	5.5	7.3	7.1	1.4	7.9	8.3	3.8	4.2*	6.1	5.6**	2.5	5.5	7.4	3.3	8.9	4.5
		(3.6-6.2)	(2.5-8.4)	(4.4-10.2)	(4.6-9.6)	(0.4-4.9)	(5.6-10.2)	(5.5-11.0)	(2.5-5.2)	(1.9-6.5)	(3.2-9.1)	(1.6-9.7)	(1.6-3.5)	(4.4-6.6)	(4.7-10.2)	(2.3-4.3)	(6.0-11.9)	(3.8-5.2)
	Male	3.4	0.9	2.0	2.3	0.7	2.1	4.2	1.9	2.6**	3.0	1.1**	5.9	0.6**	3.6	1.6***	*****	0.2
		(2.5-4.3)	(0.0-1.8)	(0.3-3.6)	(1.4-3.1)	(0.1-4.5)	(1.0-3.1)	(2.2-6.2)	(0.7-3.2)	(0.0-5.7)	(1.2-4.7)	(0.1-2.1)	(3.3-8.5)	(0.0-1.1)	(1.8-5.4)	(0.6-2.5)		(0.0-0.6)
Physical violence only	Female	17.9	33.2	19.9	26.7	43.6	21.3	18.7	18.1	12.5*	25.6	11.1	23.6	21.0	16.3	19.9	20.6	10.3
		(15.2-20.5)	(27.9-38.5)	(14.0-25.7)	(23.2-30.3)	(36.8-50.7)	(17.1-25.4)	(14.7-22.8)	(16.0-20.3)	(7.4-17.7)	(19.9-31.3)	(8.0-14.1)	(19.2-27.9)	(18.6-23.3)	(12.1-20.4)	(16.0-23.7)	(17.3-23.8)	(9.1-11.4)
	Male	20.3	39.8	34.9	32.2	37.3	32.0	24.9	31.4	26.0*	42.8	15.3	30.1	47.5	26.9	31.7	41.5	19.0
		(17.5-23.1)	(34.7-44.8)	(29.5-40.4)	(28.7-35.8)	(29.6-45.7)	(28.9-35.1)	(20.4-29.3)	(27.8-34.9)	(17.0-35.1)	(36.6-49.0)	(11.4-19.2)	(25.4-34.9)	(41.9-53.2)	(20.9-33.0)	(26.5-37.0)	(37.2-45.9)	(14.5-23.5)
Emotional violence only	Female	5.0	3.3	4.7	3.8	2.9	6.5	5.2	5.6	8.1*	4.2	3.9	6.1	3.5	0.4***	2.7	*****	4.3
		(3.7-6.3)	(1.3-5.2)	(2.6-6.8)	(1.9-5.7)	(1.3-6.6)	(4.4-8.5)	(2.9-7.5)	(4.4-6.8)	(3.4-12.8)	(2.1-6.2)	(2.5-5.3)	(3.8-8.4)	(2.8-4.3)	(0.1-0.7)	(1.8-3.7)		(3.6-5.0)
	Male	2.9	4.8	3.8	4.4	7.1	4.4	6.4	4.0	1.9**	2.5	1.5**	6.1	1.0**	2.4***	2.4	4.0	3.1
		(1.8-3.9)	(2.7-6.8)	(1.8-5.9)	(3.2-5.7)	(3.6-13.6)	(3.1-5.6)	(3.9-8.9)	(2.4-5.6)	(0.4-3.3)	(1.1-4.0)	(0.5-2.4)	(3.1-9.1)	(0.3-1.7)	(0.6-4.2)	(1.2-3.7)	(2.1-5.9)	(1.2-5.0)
Sexual violence and physical violence	Female	-	11.8	7.3	11.2	5.1	13.3	6.8	2.8	-	-	-	10.0	-	-	-	8.4	-
			(8.6-15.0)	(4.2-6.8)	(8.7-13.7)	(3.0-8.5)	(10.1-16.5)	(4.6-9.0)	(1.8-3.7)				(6.9-13.1)				(5.8-11.0)	
	Male	-	6.9	3.8	4.7	1.1	5.9	2.4	2.2	-	-	-	6.6	-	-	-	4.9	-
			(3.2-10.7)	(1.8-5.9)	(3.3-6.1)	(0.4-3.0)	(4.3-7.5)	(1.1-3.8)	(1.2-3.2)				(3.8-9.4)				(3.2-6.6)	
Sexual violence and emotional violence	Female	-	1.5	0.2	1.1	0.2	1.6	2.0	0.8	-	-	-	1.4	-	-	-	*****	-
			(0.4-2.5)	(0.0-0.5)	(0.5-1.7)	(0.1-0.7)	(0.3-2.9)	(0.8-3.2)	(0.4-1.2)				(0.5-2.3)					
	Male	-	0.6	0.1	0.6	0.8	1.1	0.5	0.2	-	-	-	0.6	-	-	-	*****	-
			(0.1-1.2)	(0.0-0.4)	(0.0-1.1)	(0.1-5.2)	(0.4-1.8)	(0.0-1.0)	(0.0-0.3)				(0.0-1.2)					
Physical and emotional violence	Female	-	8.1	8.2	6.2	14.9	12.3	5.2	5.6	-	-	-	15.2	-	-	-	*****	-
			(5.6-10.6)	(4.8-11.7)	(4.2-8.1)	(10.6-20.6)	(9.5-15.1)	(2.9-7.6)	(4.5-6.8)				(11.6-18.8)					
	Male	-	17.7	16.9	11.9	23.6	22.7	10.1	8.2	-	-	-	12.3	-	-	-	10	-
			(13.7-21.7)	(13.2-20.6)	(9.2-14.6)	(14.6-30.9)	(20.0-25.5)	(6.5-13.6)	(6.2-10.2)				(8.6-15.9)				(7.3-12.8)	
Two types of violence	Female	12.3	-	-	-	-	-	-	-	9.9*	17.0	7.8	-	10.9	6.2	11.7	-	6.3
		(10.2-14.5)								(5.6-14.2)	(13.0-21.0)	(5.4-10.2)		(9.1-12.3)	(3.7-8.8)	(9.0-14.5)		(5.5-7.1)
	Male	8.8	-	-	-	-	-	-	-	10.4*	15.7	3.3	-	8.5	6.4	8.1	-	4.1
		(7.0-10.7)								(5.6-15.1)	(11.5-19.9)	(1.7-4.9)		(5.6-11.4)	(3.8-9.0)	(5.6-10.7)		(2.3-5.8)
Sexual violence and physical and emotional violence	Female	3.2	12.8	7.0	5.5	5.2	12.4	3.0	1.8	6.1*	5.1**	3.6	11.8	2.1	1.8***	2.0	4.5	1.1
		(2.3-4.1)	(9.3-16.4)	(3.9-10.2)	(3.7-7.2)	(2.5-10.5)	(9.0-15.9)	(1.2-4.8)	(1.0-2.7)	(0.8-11.5)	(2.0-8.2)	(2.6-4.7)	(9.0-14.6)	(1.5-2.7)	(0.2-3.4)	(1.2-2.8)	(1.9-7.1)	(0.8-1.5)
	Male	0.9	8.7	7.7	3.1	3.7	7.4	2.7	1.1	1.2**	2.5**	0.2**	8.1	1.3**	****	1.1***	3.1	0
		(0.4-1.4)	(6.1-11.3)	(4.3-11.1)	(2.1-4.2)	(1.7-7.8)	(5.9-9.0)	(1.0-4.3)	(0.5-1.8)	(0.0-2.6)	(1.0-4.0)	(0.0-0.5)	(5.9-10.4)	(0.0-2.8)		(0.0-2.1)	(1.5-4.6)	
No violence	Female	56.7	23.9	45.4	38.5	26.7	24.7	50.7	61.4	59.2	42.0	68.0	29.4	57.0	67.9	60.4	50.2	73.5
		(53.0-60.4)	(18.3-29.4)	(38.4-52.3)	(33.4-43.5)	(21.1-33.1)	(20.5-28.9)	(45.1-56.3)	(58.4-64.4)	(51.6-66.8)	(35.5-48.6)	(63.5-72.4)	(23.6-35.3)	(54.1-60.0)	(62.9-72.8)	(56.7-64.0)	(46.0-54.5)	(71.8-75.2)
	Male	63.7	20.6	29.5	40.7	25.8	24.4	48.8	51.0	57.9	33.5	78.6	30.3	41.0	59.7	55.0	34.8	73.7
		(60.2-67.2)	(15.3-25.9)	(24.2-34.9)	(37.0-44.5)	(18.0-35.4)	(21.5-27.3)	(43.7-53.9)	(47.3-54.6)	(49.2-66.6)	(26.6-40.5)	(73.9-83.3)	(25.2-35.4)	(35.0-47.1)	(52.3-67.0)	(48.6-61.4)	(29.6-40.0)	(68.3-79.0)

The table summarizes the prevalence of different types of violence and multiple forms of violence experienced prior to age 18 among 18-24 year olds, by gender. These estimates are drawn from the VACS country reports. 95% confidence intervals included in parentheses below estimates. Explanation of unstable estimates are described in the VACS country reports. Author calculations for Tanzania estimates as comparable estimates were not included in the VACS country report. Rwanda sample is for 19-24 year olds.

* RSE is > 20% and < 30%

** RSE is > 30%, unreliable estimate and result should be interpreted with caution

*** RSE is > 30% and ≤ 50%, moderately unstable estimate and result should be interpreted with caution

**** RSE is > 50%, unstable estimate and estimate is suppressed

***** For Rwanda only, RSE is > 30%, unstable estimate and results is suppressed

VACS respondents are asked whether they have experienced each type of violence based on the typology laid out below. Survey respondents are asked separately about their most recent incident (defined as occurring during the last 12 months) and earliest incident of each form of violence. Because the specific timing of these events is not clear, and the total count of incidents of each type is not clearly estimable, we pool responses on both the most recent and earliest incidents to determine whether each respondent experienced each form of violence. Respondents are then asked about the type of relationship with each perpetrator for each incident, allowing us to classify school-based incidents as those where the perpetrator was a teacher, classmate or peer. Peers include siblings, schoolmates, neighbors, or strangers who are the respondents own age. We include peers since this category includes schoolmates. Survey participants that do not respond or have missing values about the perpetrator’s sex and/or their relationship are coded as missing responses for our analysis.

We use the following typology of violence for most of the countries in our sample:

1. Peer physical violence (PPV) and classmate-perpetrated PPV
2. Teacher-perpetrated non-caregiver adult physical violence (APV)
3. Sexual violence (SV) perpetrated by classmates and teachers and incidents at school for the following forms of (SV)
  (a) Unwanted sexual touching (ST)
  (b) Attempted unwanted sex (US)
  (c) Physically forced sex (FS)
  (d) Pressured sex (PS)
  (e) One or more experiences of school-related violence of any type

Because Kenya and Tanzania were among the first countries to implement the VACS, the questionnaire in these countries did not ask questions about peer physical violence, classmate physical violence, and sexual experiences with peers and teachers in the past 12 months. In these two country samples, we thus adopted the following, modified typology:

1. Teacher-perpetrated non-caregiver adult physical violence (APV)
2. Sexual violence (SV) perpetrated by teachers, for the following forms of SV:
  (a) Unwanted sexual touching (ST)
  (b) Attempted unwanted sex (US)
  (c) Physically forced sex (FS)
  (b) Pressured sex (PS)

For respondents who indicate they experienced a given type of violence, the survey also asked whether they sought services due to experiences of violence, as well as whether they were absent from school due to each type of physical or sexual violence. We thus also tabulate rates for these actions among the respondents who experienced these forms of violence.

For all of the prevalence estimates, we utilize the full sample and then also restrict our sample to only those that have ever attended school. This allows us to characterize both the overall risk to children in the general population, as well as more narrowly focusing on the risk of violence to children when they attend school. We report estimates from the full sample but the tables have both the full and restricted sample estimates. We use the VACS sample weights to ensure the prevalence estimate calculated is nationally representative.

Table 2 provides our overall sample, with gender and age statistics:

**Table 2:** Demographic information for survey samples by country.

	Honduras	Kenya	Malawi	Nigeria	Tanzania	Uganda	Zambia	Overall
Mean Age of Female Respondents	17.66	17.67	17.40	18.33	17.64	17.66	18.31	17.81
Mean Age of Male Respondents	18.41	18.26	18.19	18.17	18.03	18.20	18.16	18.21
Number of Female Respondents	2659	1467	1133	2437	1771	2645	928	13040
Number of Male Respondents	2537	1228	1029	1766	1968	3159	891	12578
Sample Total	5196	2695	2162	4203	3739	5804	1819	25618

## Results

**Fig 1:**
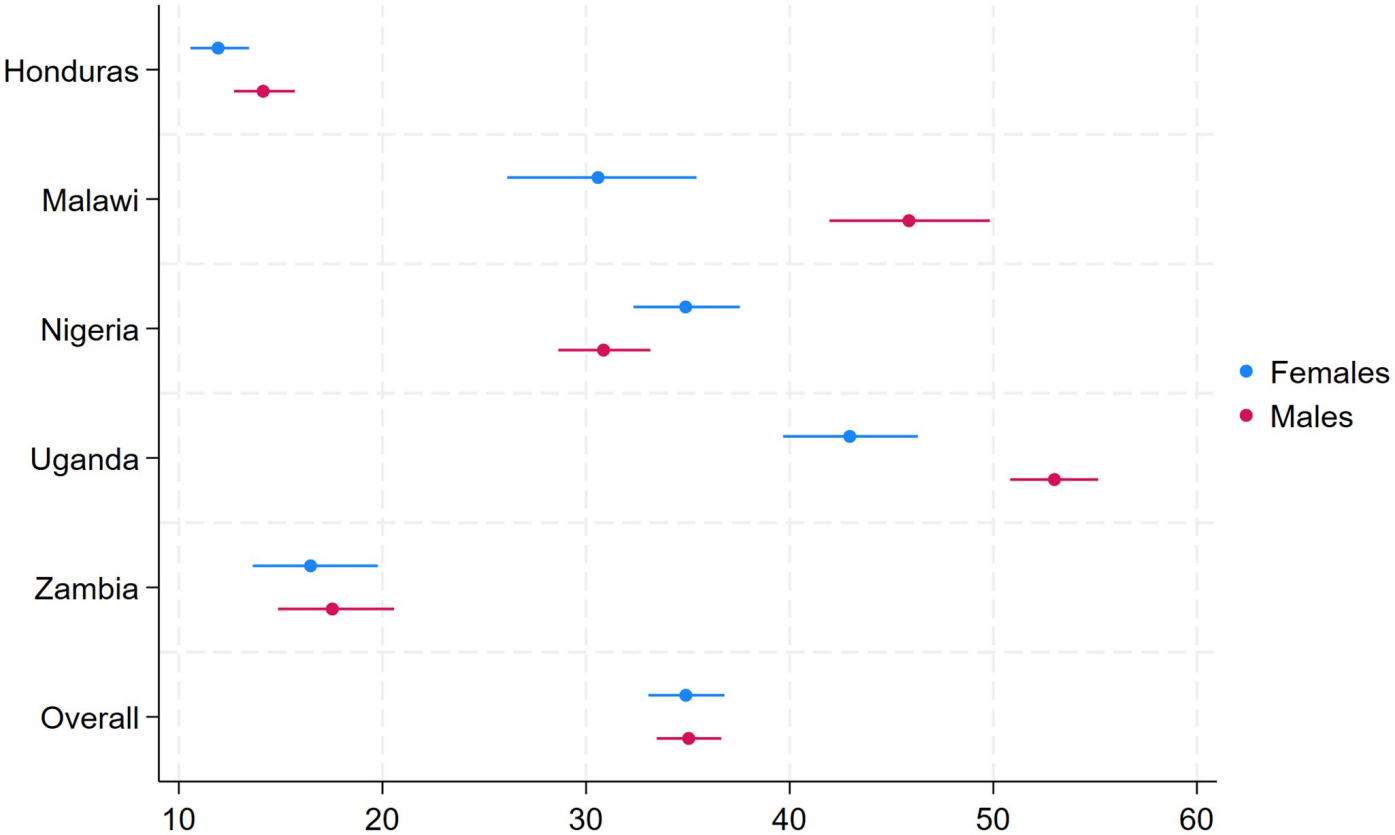
Prevalence of experiencing at least one form of physical or sexual violence for females and males. The prevalence estimates for each of the five comparable countries plus the overall prevalence estimate are provided along with 95% confidence intervals.

Table 3 provides a summary of the salient school-related violence prevalence estimates calculated for the five countries with the most directly comparable surveys (Honduras, Malawi, Nigeria, Uganda, and Zambia). Panel A of the table provides estimates for the full sample of respondents, while Panel B restricts the sample to only those who report ever attending school. This table and all following tables display 95% confidence intervals and these reflect standard errors that have been adjusted for autocorrelation within clusters (EAs) using Stata’s in-built sample survey analysis functions. The table highlights that overall rates of school-related violence are high, with more than a third of all children in the full sample experiencing at least one form of physical or sexual violence. These rates are even higher when we restrict our sample to only those who ever attended school, as the school-related violence reports are concentrated in this part of our sample. More than 40% of females who have ever attended school across all five countries report experiencing at least one form of school-related violence. Rates of school-related violence are even higher among males in all countries but one (Nigeria), driven in large part by the higher rates of peer- and classmate-perpetrated physical violence that males experience relative to females. In contrast, females experience higher rates of school-related sexual violence in three of the five countries (Malawi, Uganda, and Zambia). In Honduras males report higher rates of sexual violence than females, while in Nigeria these rates are similar between males and females. In the full sample, more than 1 in every 20 respondents reported experiencing at least form of school-related sexual violence, with considerable variation across countries. In Malawi, nearly 1 in every 10 female respondents reported experiencing school-related sexual violence, while such violence was much rarer in Honduras. In addition to Table 3, we have included Fig 1 showing the prevalence rates and associated 95% confidence intervals.

**Table 3:** Prevalence of school-related gender-based violence by gender and country.

	Respondent Category	Honduras Prevalence	Malawi Prevalence	Nigeria Prevalence	Uganda Prevalence	Zambia Prevalence	Overall Prevalence
		(95% CI)	(95% CI)	(95% CI)	(95% CI)	(95% CI)	(95% CI)
*Panel A: Entire Sample*							
Experienced one or more forms of physical and/or sexual violence	Female	11.93	30.59	34.89	42.95	16.47	33.65
		(10.57-15.70)	(26.13-49.83)	(32.33-33.16)	(39.68-55.15)	(13.63-20.57)	(32.71-34.60)
	Male	14.14	45.86	30.86	53.00	17.54	33.84
		(12.71-15.70)	(41.95-49.83)	(28.64-33.16)	(50.83-55.15)	(14.87-20.57)	(33.04-34.64)
Experienced one or more forms of sexual violence	Female	1.48	9.19	6.11	8.06	3.45	6.23
		(1.06-2.79)	(6.34-5.55)	(4.92-7.24)	(6.34-6.82)	(2.32-3.50)	(5.29-7.18)
	Male	2.14	3.89	5.96	5.77	2.22	5.36
		(1.64-2.79)	(2.71-5.55)	(4.89-7.24)	(4.87-6.82)	(1.40-3.50)	(4.56-6.16)
*Panel B: Attended School Only*							
Experienced one or more forms of physical and/or sexual violence	Female	12.11	32.07	44.42	44.63	17.07	40.29
		(10.73-15.87)	(27.41-50.94)	(41.30-36.99)	(41.26-56.03)	(14.12-21.48)	(39.13-41.45)
	Male	14.28	46.89	34.49	53.85	18.33	36.48
		(12.84-15.87)	(42.88-50.94)	(32.07-36.99)	(51.65-56.03)	(15.54-21.48)	(35.60-37.36)
Experienced one or more forms of sexual violence	Female	1.50	9.66	7.82	8.36	3.53	7.43
		(1.08-2.83)	(6.66-5.73)	(6.30-7.97)	(6.57-6.93)	(2.36-3.69)	(6.27-8.60)
	Male	2.17	4.02	6.56	5.85	2.34	5.78
		(1.66-2.83)	(2.80-5.73)	(5.39-7.97)	(4.94-6.93)	(1.47-3.69)	(4.90-6.66)

95% confidence intervals included in parentheses below estimates.

Table 4 presents prevalence estimates for physical violence perpetrated by both peers and classmates among the five countries with the most comparable surveys. In general, males more frequently report experiencing physical violence than females, with notable exceptions. Many of these rates are very high. In Malawi, more than half of all males report experiencing physical violence at the hands of a male peer (53%), with these rates nearly as high in Uganda (45%) and Nigeria (37%). Females are much less likely to experience physical violence than males (overall rates varying between 4% and 16%), although rates of females reporting physical violence by their female peers remain notable (reaching 19% in Malawi and 17% in Zambia).

**Table 4:** Peer physical violence, classmate physical violence & school absenteeism.

	Respondent Category	Honduras Prevalence	Malawi Prevalence	Nigeria Prevalence	Uganda Prevalence	Zambia Prevalence
		(95% CI)	(95% CI)	(95% CI)	(95% CI)	(95% CI)
*Panel A: Entire Sample*						
PPV by age group	Female	14.57	40.32	26.72	31.91	16.04
		(13.07-16.21)	(35.81-44.99)	(24.42-29.16)	(29.45-34.47)	(13.18-19.39)
	Male	10.59	29.99	21.50	28.76	15.81
		(9.40-11.91)	(26.18-34.08)	(19.65-23.47)	(26.33-31.32)	(13.41-18.55)
PPV perpetrated by female peer	Female	3.97	18.52	10.90	16.72	6.96
		(3.20-4.90)	(14.63-23.16)	(9.37-12.64)	(14.33-19.42)	(5.13-9.37)
	Male	3.99	1.52	1.61	2.77	1.50
		(3.23-4.91)	(0.82-2.80)	(1.14-2.26)	(2.13-3.60)	(0.80-2.78)
PPV perpetrated by male peer	Female	10.32	12.26	11.62	16.37	3.73
		(9.04-11.76)	(9.39-15.86)	(9.98-13.49)	(14.03-19.00)	(2.46-5.63)
	Male	20.99	52.58	37.16	45.14	25.24
		(19.31-22.78)	(48.55-56.57)	(34.85-39.52)	(42.99-47.32)	(22.11-28.65)
Classmate-perpetrated PPV by female classmate	Female	2.99	3.68	3.69	5.72	2.15
		(2.29-3.89)	(1.88-7.07)	(2.91-4.68)	(4.32-7.54)	(1.07-4.26)
	Male	0.60	0.00	0.18	0.52	0.15
		(0.33-1.08)	-	(0.07-0.45)	(0.32-0.85)	(0.02-1.06)
Classmate-perpetrated PPV by male classmate	Female	6.59	3.98	5.36	7.07	0.80
		(5.58-7.77)	(2.85-5.53)	(4.33-6.60)	(5.58-8.92)	(0.34-1.90)
	Male	11.52	13.99	10.58	17.25	2.79
		(10.21-12.97)	(11.46-16.97)	(9.21-12.12)	(15.68-18.95)	(1.83-4.22)
School absenteeism due to classmate-perpetrated PPV	Female	20.14	10.01	17.28	29.27	19.15
		(14.67-27.01)	(4.95-19.19)	(11.60-24.95)	(21.73-38.15)	(3.38-61.62)
	Male	14.87	12.35	11.35	26.62	4.94
		(10.52-20.61)	(6.92-21.08)	(7.67-16.48)	(22.13-31.65)	(0.64-29.41)
*Panel B: Attended School Only*						
PPV by Age Group	Female	14.53	40.52	28.71	31.99	15.89
		(13.03-16.18)	(35.97-45.24)	(26.17-31.39)	(29.50-34.58)	(13.01-19.27)
	Male	10.49	30.43	23.56	29.47	16.45
		(9.30-11.82)	(26.43-34.74)	(21.46-25.80)	(26.96-32.12)	(13.94-19.32)
PPV perpetrated by female peer	Female	3.93	18.87	12.17	17.10	7.24
		(3.16-4.87)	(14.82-23.72)	(10.37-14.22)	(14.63-19.89)	(5.34-9.74)
	Male	4.05	1.44	1.58	2.84	1.57
		(3.29-4.98)	(0.74-2.75)	(1.08-2.29)	(2.18-3.69)	(0.84-2.92)
PPV perpetrated by male peer	Female	10.47	12.61	13.56	17.00	3.80
		(9.17-11.93)	(9.60-16.38)	(11.61-15.78)	(14.57-19.73)	(2.48-5.77)
	Male	20.93	52.85	38.40	45.42	25.62
		(19.23-22.73)	(48.74-56.92)	(35.96-40.91)	(43.23-47.62)	(22.41-29.13)
Classmate-perpetrated PPV by female classmate	Female	3.03	3.86	4.64	5.93	2.23
		(2.33-3.95)	(1.98-7.42)	(3.64-5.89)	(4.47-7.82)	(1.11-4.43)
	Male	0.61	0.00	0.20	0.54	0.16
		(0.34-1.10)	-	(0.08-0.52)	(0.33-0.87)	(0.02-1.11)
Classmate-perpetrated PPV by male classmate	Female	6.69	4.18	6.72	7.35	0.84
		(5.66-7.88)	(2.99-5.82)	(5.42-8.30)	(5.80-9.27)	(0.35-1.98)
	Male	11.62	14.45	11.70	17.42	2.93
		(10.30-13.09)	(11.84-17.52)	(10.17-13.43)	(15.82-19.15)	(1.93-4.43)
School absenteeism due to classmate-perpetrated PPV	Female	20.31	10.01	17.63	29.36	19.15
		(14.79-27.22)	(4.96-19.16)	(11.85-25.41)	(21.80-38.27)	(3.53-60.55)
	Male	15.06	12.35	11.71	27.03	4.94
		(10.66-20.86)	(6.93-21.06)	(7.92-16.97)	(22.48-32.13)	(0.68-28.41)

95% confidence intervals included in parentheses below estimates.

We also find that these rates are only partly due to physical violence perpetrated by classmates, with these rates much lower overall than those reflecting peer perpetrators. This could be because this violence is perpetrated by older students from higher grades, or, in larger school settings with multiple classes, violence perpetrated by same-aged peers who are not classmates. Alternatively, such violence could be perpetrated outside of the school setting.

The harms of this physical violence are substantial. In addition to the victims’ physical pain, injuries, and psychological trauma, there is also evidence in the VACS data suggesting they suffer educational costs as well. Many of the respondents who experienced physical violence by classmates report subsequently missing school. In Uganda, these rates are nearly 30% for both females and males, suggesting an important educational impact from such violence.

In addition to physical violence at the hands of their peers, children also frequently experience physical violence perpetrated by their teachers. Table 5 shows the rates of physical violence perpetrated by teachers, as well as the rates of school absenteeism among those who experience it. Tanzania and Kenya are also included in this analysis as the surveys in these countries include questions related to teacher-perpetrated violence, and these countries exhibit some of the highest rates of such violence. In five of the seven countries, more than in 1 in 5 children experience physical violence at the hands of their teachers. Only in Zambia (13%) and Honduras (1%) is such violence much less frequent. There is considerable variation in how frequently this violence causes students to miss school, although the combined rates of violence and resulting absenteeism are particularly troubling in Uganda, where nearly 27% of females report missing school due to physical violence by their teachers.

**Table 5:** Teacher-perpetrated adult physical violence.

	Respondent Category	Honduras Prevalence	Malawi Prevalence	Nigeria Prevalence	Uganda Prevalence	Zambia Prevalence	Kenya Prevalence	Tanzania Prevalence
		(95% CI)	(95% CI)	(95% CI)	(95% CI)	(95% CI)	(95% CI)	(95% CI)
*Panel A: Entire Sample*								
Teacher-perpetrated APV by age group	Female	1.27	37.22	26.81	45.36	16.19	68.56	55.68
		(0.86-1.88)	(32.85-41.81)	(24.49-29.26)	(42.54-48.21)	(13.17-19.75)	(64.79-72.09)	(51.54-59.74)
	Male	1.50	23.07	24.03	34.20	11.12	60.72	41.27
		(1.04-2.18)	(19.79-26.70)	(21.91-26.28)	(31.56-36.94)	(9.10-13.51)	(57.14-64.18)	(37.26-45.39)
Teacher-perpetrated APV	Female	1.72	22.40	28.73	34.69	12.11	63.14	46.64
		(1.21-2.45)	(18.56-26.77)	(26.31-31.27)	(31.60-37.92)	(9.64-15.10)	(59.29-66.82)	(42.68-50.65)
	Male	1.05	37.27	21.60	44.55	14.80	65.52	49.53
		(0.70-1.59)	(33.58-41.11)	(19.62-23.71)	(42.43-46.69)	(12.28-17.73)	(62.05-68.85)	(45.22-53.85)
School absenteeism due to teacher-perpetrated APV	Female	57.44	21.11	9.33	26.84	9.17		
		(39.16-73.89)	(13.83-30.84)	(6.72-12.81)	(22.30-31.92)	(4.82-16.74)		
	Male	39.05	14.08	9.93	22.79	8.42		
		(21.32-60.24)	(10.72-18.29)	(7.39-13.22)	(20.30-25.49)	(3.98-16.92)		
*Panel B: Attended School Only*								
Teacher-perpetrated APV by age group	Female	1.29	37.55	31.89	46.03	16.69	69.42	57.83
		(0.87-1.90)	(33.13-42.19)	(29.20-34.71)	(43.18-48.91)	(13.59-20.34)	(65.65-72.95)	(53.59-61.96)
	Male	1.53	24.26	28.84	35.58	11.64	63.64	45.83
		(1.06-2.22)	(20.77-28.12)	(26.36-31.45)	(32.85-38.42)	(9.52-14.16)	(60.01-67.13)	(41.46-50.26)
Teacher-perpetrated APV	Female	1.75	23.47	36.59	36.09	12.60	65.66	51.58
		(1.23-2.49)	(19.44-28.04)	(33.61-39.67)	(32.89-39.41)	(10.03-15.70)	(61.75-69.36)	(47.33-55.81)
	Male	1.07	38.00	24.22	45.31	15.43	67.04	51.69
		(0.71-1.61)	(34.22-41.93)	(22.03-26.56)	(43.16-47.48)	(12.80-18.49)	(63.56-70.34)	(47.23-56.13)
School absenteeism due to teacher-perpetrated APV	Female	57.44	21.18	9.43	26.86	9.17		
		(39.41-73.69)	(13.88-30.94)	(6.79-12.95)	(22.32-31.94)	(4.83-16.72)		
	Male	39.05	14.26	10.14	22.99	8.51		
		(21.52-59.96)	(10.85-18.52)	(7.55-13.50)	(20.47-25.71)	(4.03-17.08)		

95% confidence intervals included in parentheses below estimates.

Although the prevalence of physical violence is quite high for both males and females, across all countries we do find that sexual violence is prevalent as well. In general, across all countries, sexual violence perpetrated by classmates is higher than teacher-perpetrated sexual violence. We find that unwanted sexual touching by classmates is high across all countries, as reported in Table 6. Rates of unwanted sexual touching reported by females vary between 0.5-5% across these countries, with such touching particularly prevalent in Uganda, Nigeria, and Malawi. Even among boys, we see comparably high rates of unwanted sexual touching.

**Table 6:** Sexual violence perpetrated by classmates and teachers.

	Respondent Category	Honduras Prevalence	Malawi Prevalence	Nigeria Prevalence	Uganda Prevalence	Zambia Prevalence	Kenya Prevalence	Tanzania Prevalence
		(95% CI)	(95% CI)	(95% CI)	(95% CI)	(95% CI)	(95% CI)	(95% CI)
*Panel A: Entire Sample*								
ST by classmates	Female	1.17	3.77	3.09	5.44	1.58		
		(0.81-1.67)	(2.18-6.44)	(2.24-4.24)	(4.16-7.09)	(0.91-2.76)		
	Male	1.68	0.95	4.00	4.14	1.59		
		(1.23-2.29)	(0.54-1.66)	(3.14-5.08)	(3.39-5.06)	(0.89-2.81)		
ST by teachers	Female	0.05	0.47	0.62	1.11	0.37	1.80	2.61
		(0.01-0.20)	(0.12-1.75)	(0.31-1.24)	(0.40-3.03)	(0.11-1.21)	(0.91-3.54)	(1.51-4.46)
	Male	0.13	0.04	0.57	0.02	0.00	0.68	0.30
		(0.05-0.32)	(0.01-0.26)	(0.27-1.17)	(0.00-0.18)	-	(0.30-1.51)	(0.10-0.92)
US by classmates	Female	0.36	4.84	1.50	1.81	1.01		
		(0.15-0.86)	(2.71-8.50)	(0.98-2.28)	(1.19-2.74)	(0.49-2.05)		
	Male	0.46	2.66	1.45	2.08	0.45		
		(0.24-0.88)	(1.67-4.20)	(0.94-2.23)	(1.56-2.76)	(0.18-1.10)		
US by teachers	Female	0.05	0.45	0.60	0.71	0.26	1.18	1.77
		(0.01-0.32)	(0.18-1.11)	(0.30-1.17)	(0.33-1.52)	(0.06-1.23)	(0.39-3.49)	(0.78-3.93)
	Male	0.03	0.00	0.24	0.03	0.00	0.48	0.48
		(0.00-0.20)	-	(0.09-0.66)	(0.00-0.19)	-	(0.21-1.09)	(0.09-2.44)
FS by classmates	Female	0.06	0.18	0.58	0.42	0.00		
		(0.01-0.44)	(0.04-0.78)	(0.30-1.09)	(0.12-1.44)	-		
	Male	0.09	0.22	0.35	0.15	0.24		
		(0.02-0.37)	(0.05-1.04)	(0.14-0.92)	(0.06-0.37)	(0.06-0.97)		
FS by teachers	Female	.	0.23	0.47	0.17	0.06	0.41	0.18
		-	(0.05-0.95)	(0.22-1.01)	(0.06-0.50)	(0.01-0.46)	(0.09-1.82)	(0.04-0.93)
	Male	.	0.00	0.04	0.00	0.00	0.09	0.08
		-	-	(0.01-0.18)	-	-	(0.01-0.61)	(0.01-0.55)
School absenteeism due to SV	Female	10.42	21.95	17.84	10.64	12.45		
		(4.17-23.70)	(9.20-43.85)	(10.13-29.51)	(5.69-19.01)	(3.77-34.04)		
	Male	6.05	3.78	4.00	7.25	5.19		
		(2.40-14.44)	(1.07-12.50)	(1.44-10.66)	(4.23-12.16)	(1.18-20.00)		
*Panel B: Attended School Only*								
ST by classmates	Female	1.18	3.96	3.92	5.66	1.58		
		(0.83-1.70)	(2.29-6.76)	(2.84-5.40)	(4.33-7.38)	(0.89-2.80)		
	Male	1.71	0.98	4.32	4.21	1.67		
		(1.25-2.33)	(0.56-1.72)	(3.39-5.47)	(3.44-5.15)	(0.94-2.96)		
ST by teachers	Female	0.05	0.49	0.79	1.15	0.38	1.87	2.89
		(0.01-0.20)	(0.13-1.84)	(0.39-1.59)	(0.41-3.16)	(0.12-1.26)	(0.94-3.69)	(1.68-4.94)
	Male	0.13	0.04	0.61	0.00	0.00	0.69	0.32
		(0.05-0.32)	(0.01-0.27)	(0.29-1.31)	-	-	(0.31-1.54)	(0.10-0.96)
US by classmates	Female	0.36	5.09	1.93	1.88	1.05		
		(0.15-0.87)	(2.85-8.92)	(1.26-2.94)	(1.23-2.85)	(0.51-2.13)		
	Male	0.47	2.74	1.65	2.09	0.47		
		(0.24-0.90)	(1.73-4.34)	(1.07-2.53)	(1.56-2.79)	(0.19-1.16)		
US by teachers	Female	0.05	0.47	0.77	0.74	0.27	1.23	1.96
		(0.01-0.33)	(0.19-1.17)	(0.39-1.51)	(0.34-1.58)	(0.06-1.28)	(0.41-3.65)	(0.87-4.34)
	Male	0.03	0.00	0.27	0.03	0.00	0.49	0.51
		(0.00-0.21)	-	(0.10-0.75)	(0.00-0.19)	-	(0.21-1.12)	(0.10-2.56)
FS by classmates	Female	0.06	0.19	0.74	0.43	0.00		
		(0.01-0.44)	(0.04-0.82)	(0.39-1.41)	(0.12-1.51)	-		
	Male	0.09	0.23	0.40	0.15	0.25		
		(0.02-0.37)	(0.05-1.07)	(0.15-1.05)	(0.06-0.38)	(0.06-1.02)		
FS by teachers	Female	.	0.24	0.60	0.16	0.07	0.43	0.20
		-	(0.06-1.00)	(0.28-1.30)	(0.05-0.52)	(0.01-0.48)	(0.10-1.90)	(0.04-1.03)
	Male	.	0.00	0.05	0.00	0.00	0.09	0.09
		-	-	(0.01-0.21)	-	-	(0.01-0.63)	(0.01-0.58)
School absenteeism due to SV	Female	10.42	21.95	17.84	10.64	12.45		
		(4.17-23.70)	(9.20-43.85)	(10.13-29.50)	(5.69-19.01)	(3.77-34.02)		
	Male	6.05	3.78	4.00	7.25	5.19		
		(2.40-14.44)	(1.07-12.50)	(1.44-10.66)	(4.23-12.16)	(1.19-19.98)		

95% confidence intervals included in parentheses below estimates.

Forced sex perpetrated by classmates and teachers is also higher in some countries than others. In Nigeria, we find the highest prevalence of forced sex perpetrated against females and males. We find that 0.6% of females and 0.4% of males experience forced sex perpetrated by classmates, and 0.4% of females and 0.04% of males experience this by teachers. Lastly, we also find that females report higher levels of school absenteeism due to sexual violence than males across the five most comparable countries. School absenteeism among females ranges from 10-22% while for males this ranges from 4%-7%. More females report experiencing sexual violence than males and so we anticipate higher rates of school absenteeism from females. However, another driving factor for the lower school absenteeism rates for males may be due to negative stigma related to sexual violence.

## Discussion

This study has sought to quantify the extent and impact of school-related violence across several countries in Sub-Saharan Africa and Central America. We did this utilizing the nationally representative VACS data to analyze several forms of physical and sexual violence. With this data, we are able to disaggregate the violence prevalence by gender and perpetrator type. Our study highlights three important findings.

The first finding is that many children experience physical violence at school, much more than might be commonly believed or otherwise reported. The prevalence of physical violence is high across all countries analyzed and this is especially true of males. Males are victims of higher rates of physical violence and are also more likely to be perpetrators of physical violence. When we look at the overall prevalence rate of school-related violence including both physical and sexual violence, we see that about a third of females and males experience school-related violence.

The second finding is that the perpetrators of this violence include both other students and teachers, highlighting that school-related violence is not perpetrated by only one type of person. Physical violence perpetrated by male and female classmates against different gendered classmates (i.e., male against female classmates and female against male classmates) occurs; however, this occurs less frequently than same-gender violence. Additionally, across all countries analyzed, we find that male-against-female classmate violence is higher than female-against-male classmate violence. Violence in schools, however, is not only perpetrated by classmates. Teachers also perpetrate physical violence against students and this violence is directed at both males and females. In some countries, including Nigeria and Honduras, the prevalence of physical violence perpetrated by teachers is higher for females than for males.

Our final finding is that this violence has many potential impacts, but one of the clearest ones is higher rates of absenteeism from school, a pattern we particularly see among females. For almost all of the countries we analyze, females report higher rates of absenteeism regardless of whether this is for physical or sexual violence. Although males experience more physical violence than females, females report high rates of school absenteeism. From our analysis, it is unclear whether this is due to stereotypes about masculinity in which males continue to show up to school despite physical altercations, or whether the types of physical violence females experience are more severe. When restricting the sample to sexual violence, there is a higher prevalence of sexual violence experienced by females and a corresponding increase in school absenteeism. When taken together, these three findings highlight the multifaceted nature of school-related violence.

## Conclusion

Our study has sought to quantify this problem in several African and Central American countries and highlights that this is a significant challenge children and young adults face. We find that males and females experience physical and sexual school-related violence and that this is perpetrated by both classmates and teachers. Students who are affected by school-related violence also report higher levels of school absenteeism. As programs and policies are introduced to end school-related violence and reach Sustainable Development Goals 4 and 5, understanding the complex nature of violence in schools is necessary. This study provides a glimpse into this problem but more data should be collected to ensure we continue to monitor and quantify the incidence and effects of school-related violence.

Ultimately, our study allows us to provide general prevalence rates for physical and sexual violence perpetrated within schools. Although this is an important first step of quantifying the problem, there is more that needs to be done. The VACS surveys are limited in their scope and do not contain enough information for us to measure the impacts of violence on educational and learning outcomes. Though we have data on school absenteeism, and have shown that absenteeism increases with violence experiences, the VACS do not contain information about learning outcomes. It is reasonable to assume that more frequent school absenteeism among victims of school-related violence leads to worse learning outcomes. However, without the accompanying data, we do not know the magnitude and long-term effects of violence on educational outcomes. Further research that combines prevalence estimates with data on learning outcomes will push this research agenda forward, allowing for a holistic view of the impact of violence in schools.

In addition to educational and learning costs, we anticipate there are additional medical costs associated with injuries sustained by males and females. These particular costs are also unknowable solely from the VACS data. Linking the VACS data with other studies that capture this information would allow further investigation into the medical costs from school violence. As additional data is brought to bear on school violence and the associated costs, whether educational or medical, policies and programs can be better targeted in ways that not only reduce school related violence but also ameliorate longer impacts of violence.
